# A 10-year cesarean section rate analysis in a Brazilian referral maternity hospital using the Robson's ten group classification system

**DOI:** 10.61622/rbgo/2025rbgo51

**Published:** 2025-07-15

**Authors:** Maria Laura Alves de Melo Silva, José Paulo de Siqueira Guida, Giuliane Jesus Lajos, Maria Laura Costa, Adriana Gomes Luz

**Affiliations:** 1 Universidade Estadual de Campinas Departamento de Tocoginecologia Campinas SP Brazil Departamento de Tocoginecologia, Universidade Estadual de Campinas, Campinas, SP, Brazil.

**Keywords:** Cesarean section, Premature birth, Infant, newborn, Pregnancy, Robson classification

## Abstract

**Objective:**

The Robson Ten Group Classification System categorizes women into groups based on obstetric characteristics. For each group there is a suggested cesarean section rate. Robson Ten Group Classification System allows for surveillance and evaluation of increasing cesarean section rate. This study aimed to evaluate deliveries in a Brazilian referral maternity hospital in the last decade using the Robson Ten Group Classification System.

**Methods:**

This was a retrospective cross-sectional study performed in a referral hospital, analyzing deliveries from January 2009 to August 2022. Women were classified into Robson's 10 groups based on electronic medical charts. Overall rates per year and cesarean section rate within each group were calculated and compared.

**Results:**

There was an increasing cesarean section rate over time (46.23% in 2009 vs 62.99% in 2022) in all groups. Groups 1-4, 5 and 10 had a significant increase. Among Groups 1-4 cesarean section rate increased from 34.06% to 38.59% (PR 1.132, CI 1.007-1.274), group 5 from 67.66% to 83.53% (PR 1.234, CI 1.151-1.323) and group 10 from 51.55% to 60% (PR 1.163, CI 1.017-1.332). In global analysis, groups 1-4 corresponded to 57.3% of included cases and its relative contribution to cesarean section rate was 31.6%, while group 5 represented 18.9% of cases and its relative contribution to cesarean section rate was 28.5%.

**Conclusion:**

Groups 1-4 and 5 contributed significantly to cesarean section rate in our analysis and group 10 (preterm birth) also had a major impact, considering the high risk setting. Cesarean section rate increased over time. Groups 1, 2, 5, and 10 contribute significantly to such an increase.

## Introduction

The Robson Ten Group Classification System (RTGCS) was implemented by the World Health Organization (WHO) in 2015.^(1)^ The RTGCS categorizes women according to key obstetric characteristics (gestational age, parity, previous cesarean delivery, onset of labor, fetal presentation and number of fetuses) in ten different groups (Chart 1). Those groups are mutually exclusive and completely inclusive. The implementation of the RTGCS provides data regarding obstetric characteristics of population, such as its distribution through the ten groups and the cesarean section rate (CSR) in each group, as well as the overall CSR. It allows comparison of population characteristics and CSR among time and among different facilities, regions or countries.^(1)^

**Chart 1:** Description of Robson's ten group classification system

Group	Description
1	Nulliparous, singleton, cephalic, ≥37 weeks’ gestation, in spontaneous labor.
2 2a 2b	Nulliparous, singleton, cephalic, ≥37 weeks’ gestation, induced labor or cesarean section before labor. Nulliparous, singleton, cephalic, ≥37 weeks’ gestation with induced labor. Nulliparous, singleton, cephalic, ≥37 weeks’ gestation with cesarean section before labor.
3	Multiparous (excluding previous cesarean section), singleton, cephalic, ≥37 weeks’ gestation, in spontaneous labor
4 4a 4b	Multiparous (excluding previous cesarean section), singleton, cephalic pregnancy, ≥37 weeks’ gestation, induced labor or cesarean section before labor. Multiparous (excluding previous cesarean section), singleton, cephalic pregnancy, ≥37 weeks’ gestation with induced labor. Multiparous (excluding previous cesarean section), singleton, cephalic pregnancy, ≥37 weeks’ gestation with cesarean section before labor.
5	Previous cesarean section, singleton, cephalic, ≥37 weeks’ gestation.
6	All nulliparous with a single breech.
7	All multiparous with a single breech (including previous cesarean section).
8	All multiple pregnancies (including previous cesarean section).
9	All women with a single pregnancy in transverse or oblique lie (including those with previous cesarean section).
10	All singleton, cephalic, <37 weeks’ gestation pregnancies (including previous cesarean section).

**Source:** Adapted from Robson (2001).^(2)^

Previous studies in Brazil using the RTGCS indicated an increase in CSR among women in groups 1 to 4 (nulliparous or multiparous women with full term pregnancies, with cephalic fetus, without previous cesarean section), in which expected CSR are low. This trend will impact, in the future, the proportion of women in group 5 (women with full term cephalic pregnancy, with at least one previous cesarean section). This group is of special interest because its CSR is high and its prevalence is growing.^(3,4)^ This trend is also observed in other countries.^(5,6)^

Cesarean section is a life-saving procedure, specially when indicated due to some maternal or fetal conditions; however, experts defined that populational proportions higher than 15% are not related with better maternal or perinatal results. The evaluation of obstetric care using RTGCS helps a better understanding of changes in population characteristics and CSR.^(1)^

The aim of this study was to evaluate all deliveries in a Brazilian referral maternity hospital between 2009 and 2022, according to the RTGCS and analyze trends in overall CSR and the role of each one of the groups in such numbers.

## Methods

This was a retrospective cross-sectional study, including all deliveries that occurred in a high complex obstetric service between January 2009 to August 2022. The variables parity, previous cesarean section, gestational age, fetal presentation, onset of labor and mode of delivery were obtained. Data were extracted from the electronic medical charts and extensively revised for consistency. Based on the variables obtained, parturients were classified into Robson's 10 groups (Chart 1). The frequency and rate of cesarean sections were obtained in each group, as well as the cesarean rate in each of the evaluated years. Cesarean section indication was also analyzed.

The setting is located in Southeast Brazil and it covers a population of 1,000,000 inhabitants. It counts with 18 beds in obstetric ward and all deliveries are paid by the local government.

Data analysis considered risk groups for cesarean section: groups 1 to 4 (low risk groups for cesarean section), group 5 (influence of a previous cesarean section in delivery route), which was subdivided in group 5.1 (women with one previous cesarean section) and group 5.2 (women with 2 or more cesarean section rate), groups 6 to 9 (with expected high cesarean section rates) and group 10. Among preterm birth (group 10), gestational age is key in deciding on management and delivery route, which is why our analysis opted for the division into group 10.1 (early preterm - gestational age from 24 to 33 weeks and 6 days) and group 10.2 (late preterm - gestational age from 34 to 36 weeks and 6 days).

A decrease in the total number of deliveries was observed among years in our referral maternity. In order to understand the most significant differences, we further compared the initial 2 years with the latest 2 year-period. It is important to reinforce that the last 2 years data was also influenced by the COVID-19 pandemic. Prevalence ratio and confidence intervals were calculated between two periods: 2009 and 2022. P-value was obtained through Qui Square Test, with a significance level of < 0.05. Data were evaluated using EpiInfo 7.2 software.

This study was approved by the Ethics Board of the University of Campinas (5404) under the *Certificado de Apresentação de Apreciação Ética* (CAAE) number #15196419.4.0000.5404. This is a retrospective study based on medical chart review, with deidentified data, without clinical intervention and without direct patient contact, therefore the informed consent was waived. All the principles defined in the Declaration of Helsinki and Resolution 466/12 of the National Health Council were respected.

## Results

Overall, 30,040 deliveries were considered with a 48.09% CSR. Among the periods analyzed, the CSR increased from 46.23% in 2009 to 62.99% in 2022 (p<0,001). There was a marked decrease in total number of deliveries from 2018 (2232 deliveries and CSR 51.34% in 2018) until 2022 (308 deliveries and CSR 62.99% in 2022) in the considered setting (Table 1).

**Table 1:** Overall distribution of women in Robson's ten group classification system and cesarean sections in a Brazilian setting

Robson Group	Number of cesarian section in group	Number of women in group	Group size (%)	Group CS rate (%)	Absolute group contribution to overall CS rate (%)	Relative contribution of group to overall CS rate (%)
1 to 4	5489	17349	57.75	31.64	18.27	37.87
5	4144	5632	18.75	73.58	13.79	28.59
5.1	2703	4266	14.20	63.36	9.00	18.65
5.2	1441	1465	4.88	98.36	4.80	9.94
6 to 9	2828	3253	10.83	86.94	9.41	19.51
10	2035	3806	12.97	53.47	6.77	14.04
10.1	636	1176	3.91	54.08	2.12	4.39
10.2	1399	2630	8.75	53.19	4.66	9.65
Total	14496	30040	100	48.26	–	100

The global distribution of women in the RTGCS is presented in table 1. In this study, groups 1-4 corresponded to 57.7% of included cases and there was a relative contribution to CSR rate of 37.8%. Group 5 (n=4144) represented 18.7% of cases, however, its relative contribution to CSR was 28.5%. Group 5.1 corresponded to 14.2% of included cases, however, its relative contribution to CSR was 18.65%. Groups 1 to 4 (n=5489) and 5 (n=4144) were the most important contribution to overall cesarean section rate, which was 48.26% in the period analyzed (Table 2).

**Table 2:** Frequency of cesarean rates among Robson's ten group classification in a Brazilian setting compared between two periods: 2009-2010 and 2021-2022

Robson's group	2021-2022 (n=681) (%)	2009-2010 (n=2468) (%)	PR (CI)	p-value
1 to 4	38.59	34.06	1.132 (1.007-1.274)	0.0432
5	83.53	67.66	1.234 (1.151-1.323)	<0.001
5.1	76.4	58.24	1.311 (1.184-1.452)	<0.001
5.2	100	97.05	1.030 (1.007-1.053)	0.200
6 to 9	90.24	87.25	1.034 (0.973-1.098)	0.3062
10	60	51.55	1.163 (1.017-1.332)	0.0361
10.1	64.52	53.66	1.202 (0.960-1.054)	0.1310
10.2	57.89	50.69	1.142 (0.964-1.351)	0.1390

Among years analyzed (2009 to 2022), comparison between the first 2 years (2009-2010) and the last 2 years (2021-2022) showed increased cesarean rates in all groups. There was a statistical significance for all groups, except groups 6-9, as presented in table 2. For group 5, there was a CSR increase from 67.66% to 85.53% [PR 1.234 (1.151-1.323); p-value <0.001]. Group 5.1 showed this same increase in CSR, from 58.24% to 76.4% [PR 1.311 (1.184-1.452); P-value <0.001]. Indication for cesarean section is not among the considered criteria for the RTGCS because of its challenge and subjective aspect, depending on interpretation. Nevertheless, it is interesting to describe such indications as complementary data. Considering that numerical drop of deliveries has been noticed since 2018, it was chosen to analyze cesarean section indications in the institution's electronic chart for the last 5 years. Main indications for cesarean section were fetal distress, repeated cesarean section (more than 2 previous cesareans) and maternal request.

## Discussion

The global increase in cesarean section rates raises concern in healthcare services, due to short-term risks (both maternal and fetal) associated with performing the surgical procedure without medical indication, and also because of long-term impacts on the reproductive health of these patients.

In the Brazilian context, there is an alarming cesarean section rate, exceeding 50% overall,^(3,7–9)^ but potentially accounting for 80-90% of births in the private healthcare sector. In our sample, which is a high-risk obstetric service, public and teaching hospital, it was also observed a progressive increase in the cesarean section rate in recent years, with major increases in CSR in the last 5 years. This trend of a higher cesarean section rate has also been identified in other teaching hospitals in Brazil and around the world.^(8–12)^ However, there are high-income services worldwide that do not show the same trend, even when they are centers that manage high-risk pregnancies.^(13)^

Concerning our facility, the total birth rate decreased over the years and it can be linked to many reasons. There was a decrease in Brazil's birth rate, from 15.822 births (per 1000 people) in 2009 to 13.059 births (per 1000 people) in 2022.^(14)^ Furthermore, both low and high risk pregnancies were referenced to our obstetric service until 2020, but, in the last 2 years, low risk pregnancies were predominantly managed by other services, which has led to an overall drop in the number of deliveries in our service, and increase in proportion of high risk parturients.

After evaluating women into the RTGCS, it was observed a greater contribution to CSR from groups 1-4 and group 5. This was also noted in other Brazilian settings.^(2,7,9,10)^ On a global scale, there is usually a greater contribution from group 5 to the CSR, however, groups 1, 2, and 10 also stand out.^(4,11,13,15)^

In our analysis, group 5 included 18.94% of our sample, which is similar to other Brazilian studies (in which 19% of women belong to group 5).^(3)^ Induction of labor is an option for women in our facility, including those with one prior cesarean section. This is important in the Brazilian setting, in which CSR are increased among high-risk pregnancies in the public health system (once in the private health system there's a very high overall CSR).^(3,4)^

It is known that hospitals that manage high-risk pregnancies can experience an impact in the cesarean section rate, especially in group 10. Preterm birth rate in Brazil is around 11%, which can be spontaneous (spontaneous onset of labor or premature preterm rupture of membranes) or medically indicated (when providers decide to interrupt pregnancy, through labor induction or cesarean section before labor), which was similar in our analysis (12.97% of patients belonged to group 10).^(16–18)^ COVID-19 pandemic influenced the last 3 years of our analysis, once there was a numerical drop in the number of deliveries and an increase in CSR. Group 10 increased its CSR, especially 10.1 (early preterm), which may be linked to COVID-19 infection among the pregnant population.

When analyzing indications for cesarean section in our facility, it was observed that fetal distress is the main reported contributing factor, followed by having 2 or more previous cesareans and maternal request, which is a rising rate of cesarean indication in other contexts.^(19,20)^ Comparing 2009-2010 to 2021-2022, there was an increase in CSR from 67.66% to 83.53%. High overall CSR (above 60%) and significant contribution of groups 1-4 to the CSR in our service is complex and linked to many influencing factors, but it may demonstrate missed opportunities for vaginal deliveries, especially considering our context of teaching hospital. In other scenarios, there are other influencing factors on these rates, such as the payment source for delivery (public or private), as well as the timing of delivery (day or night shift) and whether the service performs cesarean section upon maternal request or not.^(7,9–11,13,21,22)^

There are several limitations in our analysis. Medical records have migrated over time from a predominantly handwritten scenario to a digital system, so some information was lost in this process. Also, data was analyzed retrospectively, using medical charts heterogeneously written (especially before the digital system was implemented). It was not possible to classify women into Robson groups 1, 2, 3 and 4, as medical records prior to 2017 didn't contain information about whether labor was induced or spontaneous (1 or 2; 3 or 4) and if cesarean section happened before labor or patient was submitted to labor induction (2a or 2b; 4a or 4b). Therefore, it was chosen to divide the population based on their risk of having a cesarean section as an outcome. Nevertheless, due to the large number of patients analyzed, we were able to observe temporal changes and the influence of the Robson group on cesarean section rates.

## Conclusion

Preventing the first cesarean section appears to be a relevant measure in this context, but further studies are needed to better understand and propose measures aimed at reducing CSR. At healthcare service level, implementation of cesarean section monitoring systems and policies that encourage vaginal delivery is crucial, once even small annual increases in cesarean section rate can lead to a substantial long-term elevation. Therefore, population health education, specially for women in reproductive age, may contribute to reducing cesarean sections based on maternal request. In summary, the overall CSR was 48.09% with a significant increase over time. There was a significant contribution from groups 1, 2, 5, and 10 to the cesarean section rate. Group 5 was linked to a higher risk of repeated cesarean section. Prospective surveillance in the healthcare system and population health education might contribute to reducing CSR, but further studies are necessary to assess the impact of such interventions.
